# Predictors of three-month mortality among hospitalized older adults in Togo

**DOI:** 10.1186/s12877-020-01907-y

**Published:** 2020-11-26

**Authors:** Fifonsi Adjidossi Gbeasor-Komlanvi, Martin Kouame Tchankoni, Akila Wimima Bakoubayi, Matthieu Yaovi Lokossou, Arnold Sadio, Wendpouiré Ida Carine Zida-Compaore, Mohaman Djibril, Mofou Belo, Amegnona Agbonon, Didier Koumavi Ekouevi

**Affiliations:** 1 Département de Santé Publique, Université de Lomé, Faculté des Sciences de la Santé, Lomé, Togo; 2 Centre Africain de Recherche en Epidémiologie et en Santé Publique, Lomé, Togo; 3 Centre Hospitalier Universitaire Sylvanus Olympio, Pavillon Militaire, Lomé, Togo; 4 Programme National de Lutte contre les Maladies Chroniques, Lomé, Togo; 5 Université de Lomé, Laboratoire de Physiologie-Pharmacologie, Lomé, Togo

**Keywords:** Ageing, Mortality, Hospital, Associated factors, Togo

## Abstract

**Background:**

Assessing hospital mortality and its predictors is important as some of these can be prevented through appropriate interventions. Few studies have reported hospital mortality data among older adults in sub-Saharan Africa. The objective of this study was to assess the mortality and associated factors among hospitalized older adults in Togo.

**Methods:**

We conducted a prospective cohort study from February 2018 to September 2019 among patients ≥50 years admitted in medical and surgical services of six hospitals in Togo. Data were recorded during hospitalization and through telephone follow-up survey within 90 days after admission. The main outcome was all-cause mortality at 3 months. Survival curves were estimated using the Kaplan-Meier method and Cox regression analyses were performed to assess predictors of mortality.

**Results:**

The median age of the 650 older adults included in the study period was 61 years, IQR: [55–70] and at least one comorbidity was identified in 59.7% of them. The all-cause mortality rate of 17.2% (95%CI: 14.4–20.4) and the majority of death (93.7%) occurred in hospital. Overall survival rate was 85.5 and 82.8% after 30 and 90 days of follow-up, respectively. Factors associated with 3-month mortality were the hospital level in the health pyramid, hospitalization service, length of stay, functional impairment, depression and malignant diseases.

**Conclusion:**

Togolese health system needs to adjust its response to an aging population in order to provide the most effective care.

**Supplementary Information:**

The online version contains supplementary material available at 10.1186/s12877-020-01907-y.

## Background

The aging of our societies is both one of the greatest achievements and challenges in all regions of the world [1]. Most developed countries already have a large population of older adults, with 20.0% of the population over 60 years of age, and this proportion is expected to increase to more than 30.0% over the next four decades [2]. In developing countries, only 10.0% of the population is currently aged 60 years and older, but this proportion is rapidly increasing [2]. Unlike developed countries where the transition to aging took place over at least a century, developing countries are expected to achieve almost similar levels of population aging by mid-century [2].

Longer life expectancy around the world affects not only the personal, social and economic lives of individuals, but also the health care system. The increasing number of older adults and the inherent complexity of their health has resulted in increased demands on the health care system [3, 4], including acute hospitalization, outpatient clinical services, home visits, and long-term and palliative care [5]. In developed countries, it is estimated that older adults use up to four times more health care services [6] and that they are three times more likely to be hospitalized than younger groups [7].

Hospitalization of older adults constitutes a challenging issue. Indeed, older adults are at higher risk of complications such as cognitive impairment, delirium, functional decline, and they often require longer lengths of stay than younger patients with the same diagnoses [8]. Also, high mortality rates have been reported among hospitalized older adults, ranging from 5% during hospital stay to 20–30% within 1 year following hospital discharge [7]. In the literature, main risk factors for hospital mortality among older adults are age, length of stay [9, 10], health conditions such as cardiovascular diseases, diabetes, stroke, cancer, and lung diseases [10], and geriatric syndromes such as fall, inappropriate drug use, malnutrition, frailty, functional impairment, and depression [10–17]. Assessing these predictors is important as some of them can be prevented through appropriate interventions. Besides, hospital mortality rate (in-hospital or post-discharge) is widely used to assess hospital performances [18, 19].

In sub-Saharan Africa (SSA), few studies have reported hospital mortality data among older adults. Hospital mortality rates in these studies ranged from 15.1% in South Africa [9] to 22.0% in Nigeria [13] . In Togo, the proportion of older adults ≥60 years is expected to increase from 5.2% in 2010 to 6.7% by 2030 [20]. Several programs have been initiated by the government to provide social support and health care resources to the older populations. To be efficient, these efforts must be based on evidence, including data on morbidity and mortality in older adults. However, to our knowledge, no study has reported mortality rates among older adults in Togo. Thus, the objective of this study was to estimate survival in the 3 months following hospitalization and its associated factors in older adults admitted to hospitals in Togo.

## Methods

### Study design and settings

This prospective cohort study was part of the Study of Hospitalized Older adults in Togo (SHOT) which aimed to describe comorbidities and 12-month post-hospitalization outcomes among hospitalized older adults aged ≥50 years in Togo. Togo is a country of West Africa which covers an area of 56,800 Km^2^ with an average density of 145 inhabitants per square kilometer. The population was 7.89 million in 2018, of which 50.2% are women [21, 22]. Most of the population is young (60% of Togolese are under 25 years of age), and lives in rural areas (62%) [21].

Togo’s health system has a three-level pyramid structure: central, intermediate and peripheral levels. For each level, there are administrative and healthcare delivery components. Regarding health care for older adults, no geriatric wards are available in the country.

The present study was carried out from February 2018 to September 2019 in the medical and surgical wards of the three main tertiary level hospitals and three hospitals from the secondary level of the health pyramid.

### Study population

The study was conducted in all three hospitals of the tertiary level of Togo’s health system. We also selected the hospitals where medical students were doing their internship and where the directors gave their authorization to conduct the study. All patients aged 50 ≥ years who were hospitalized in adult medical and surgical wards of selected hospitals during the study period were eligible to participate in the study. Exclusion criteria were refusal of older adults or their key informants to provide consent for the study or unavailability of a phone number for the follow-up survey.

### Data collection and procedures

Data collection was carried out in two steps: face-to-face interview at baseline during hospitalization and telephone interviews at one (M1) and 3 months (M3) after hospital discharge. Baseline survey was conducted by ten final-year medical students who received a one-day training on the interview questionnaire. All eligible older adults and their key informants were informed about the study procedures and invited to participate in the study. After informed consent approval, medical students recorded sociodemographic data (age, sex, marital status, education level, monthly income, availability of health insurance, living conditions) and clinical data (date of hospitalization, comorbidities, functional status within 2 weeks prior to hospitalization, diagnoses during hospitalization, nutritional status). Data collected from older adults and their key informants were subsequently completed using medical records.

Follow-up surveys were completed at M1 and M3 by telephone. The survey was conducted by two trained medical students who collected the following data at each encounter: whether they could reach the older adult or the key informant, dates of follow-up and discharge from hospital, date of death (from key informant), number of falls and rehospitalizations, and functional status.

The questionnaire used for this study is available in the Supplementary File 1 (S1).

### Outcome and covariates

The main outcome was overall mortality (all causes of death) at 3 months, whether the death occurred during hospitalization, or at home after discharge. Deaths were ascertained by review of the local obituaries and/or from key informant during telephone interview at M1 and M3. The first day of survival was recorded as the date of admission and the final follow-up was done 90 days after.

In order to assess mortality risk factors, sociodemographic and clinical data were collected as listed above. Functional status was assessed for each basic activity of the Katz index of independence in activities of daily living (ADL) (bathing, dressing, transferring from a chair, going to toilet, continence and feeding) [23]. Each item of the ADL index can obtain a score of zero (0) or (1). A score > 4 indicates less impaired or full function; 3–4 indicates moderate impairment and ≤ 2 indicates severe functional impairment. Nutritional status was assessed using the body mass index (BMI), the Mini-Nutritional Assessment Short-Form (MNA-SF) [24], and serum albumin levels. BMI was calculated as the ratio between weight in kilograms and squared height in meters (kg/m^2^). The MNA-SF is a six-item screening tool for nutritional status with a maximum score of 14 points. Albumin levels were recorded in patients’ medical file whenever available. For the present study, we defined malnourished older adults as those who had BMI < 18.5 kg/m^2^ and/or MNA-SF score ≤ 12 and/or serum albumin < 35 g/l. For the assessment of depression, we used the Mini Geriatric Depression Scale (Mini GDS) which is composed of four items [25]. Each item was worth one point and older adults with a score ≥ 1 were considered as being depressed.

### Statistical analysis

Descriptive statistics were performed and results were presented with frequency tabulations and percentages for categorical variables. Quantitative variables were presented as medians with their interquartile range (IQR). Comparisons of medians were performed using Wilcoxon-Mann-Whitney test and proportions were compared using Chi-square test or Fisher’s exact test. Survival curves were estimated using the Kaplan-Meier method and comparisons of survival curves were performed using Log-rank tests. To assess factors associated with 3-month all-cause mortality among hospitalized older adults, Cox regression analyses were performed. In the univariable regression, variables with a *p-*value < 0.20 were fitted into the multivariable analyses. A backward procedure approach was performed for selection of variables and hazard ratios (HR) were reported with corresponding 95% confidence interval (95%CI). All analyses were performed using Stata (Stata™ 11.0 College Station, Texas, USA) and R® softwares. The significance level was set at 5%.

### Ethical considerations

All eligible older adults and their key informants received detailed information about the study purpose and procedures, voluntary participation, potential risks and protections. Recruited patients or their key informants provided signed informed consent prior to the administration of the questionnaire. This study was approved by the “Comité de Bioéthique pour la Recherche en Santé (CBRS)” (Bioethics Committee for Health Research) from the Togo Ministry of Health (n°09/2018/CBRS).

## Results

A total of 659 older adults were recruited for the SHOT project. A telephone number was not available for nine older adults or their key informant and they were not included in the present study.

### Baseline socio-demographic characteristics

The median age of the 650 older adults who were included during the study period was 61 years, IQR: [55–70]. Among them, 56.8% were male, 65.5% were married or living with a partner, and only 13.8% had reached university level. Of the 650 participants, 18.8% had a monthly income ≥150 euros and 24.5% had health insurance (Table 1).

**Table 1:** Baseline socio-demographic characteristics of hospitalized older adults in Togo in 2019 (*N* = 650)

	Total (***N*** = 650)	< 60 years (***N*** = 274)	≥ 60 years (***N*** = 376)	*p*-value
**Age (years)**				
Median (IQR)	61 (55–70)	55 (52–56)	68 (63–72)	< 0.001*
**Sex, n(%)**				0.031**
Male	369 (56.8)	169 (61.7)	200 (53.2)	
Female	281 (43.2)	105 (38.3)	176 (46.8)	
**City of residence, n(%)**				0.593**
Lomé	379 (58.3)	164 (59.9)	215 (57.2)	
Other cities	190 (29.2)	77 (28.1)	113 (30.0)	
MD	81 (12.5)	33 (12.0)	48 (12.8)	
**Marital status, n(%)**				0.008**
Married/Living with a partner	426 (65.5)	196 (71.5)	230 (61.2)	
Widow	176 (27.1)	45 (16.4)	131 (34.8)	
Living alone	45 (6.9)	32 (11.7)	13 (3.5)	
MD	3 (0.5)	1 (0.4)	2 (0.5)	
**Education level, n(%)**				< 0.001**
No formal education	178 (27.4)	45 (16.4)	133 (35.4)	
Primary school	167 (25.7)	66 (24.1)	101 (26.9)	
Secondary school	200 (30.8)	128 (46.7)	72 (19.1)	
University	90 (13.8)	29 (10.6)	61 (16.2)	
MD	15 (2.3)	6 (2.2)	9 (2.4)	
**Main source of monthly revenue, n(%)**				< 0.001**
None	33 (5.1)	8 (2.9)	25 (6.6)	
Financial aid	190 (29.2)	45 (16.4)	145 (38.6)	
Pension fund	109 (16.8)	20 (7.3)	89 (23.7)	
Income generating activity	296 (45.5)	189 (69.0)	107 (28.4)	
MD	22 (3.4)	12 (4.4)	10 (2.7)	
**Monthly income (euros), n(%)**				0.648**
< 53	146 (22.5)	64 (23.4)	82 (21.8)	
[53–150[	346 (53.2)	141 (51.4)	205 (54.5)	
≥ 150	122 (18.8)	55 (20.1)	67 (17.8)	
MD	36 (5.5)	14 (5.1)	22 (5.9)	
**Health insurance, n(%)**				0.930**
No	485 (74.6)	201 (73.4)	284 (75.5)	
Yes	159 (24.5)	68 (24.8)	91 (24.2)	
MD	6 (0.9)	5 (1.8)	1 (0.3)	
**Living conditions**^a^				
**Alone, n(%)**	58 (8.8)	28 (10.1)	30 (7.8)	0.393**
**With spouse, n(%)**	369 (56.0)	169 (61.2)	200 (52.2)	0.070**
**At children’s place, n(%)**	492 (74.7)	203 (73.6)	289 (75.5)	0.832**
**Host family, n(%)**	7 (1.1)	3 (1.1)	4 (1.0)	0.996***
**Other, n(%)**	47 (7.1)	18 (6.5)	29 (7.6)	0.874**

*Wilcoxon test; **Chi square test; ***Fisher test; ^a^Multiple situation is possible

### Clinical characteristics

The median length of stay was 17 days, IQR [8–32]. Almost a quarter (24.0%) of older adults were hospitalized in a surgical service and 87.1% were admitted to a hospital of the tertiary level in the Togo health pyramid. At least one comorbidity was identified in 59.7% older adults and those aged ≥60 years had more than 2 comorbidities compared to those < 60 years (25.3% vs 14.2%; *p* < 0.001). Past history of hypertension (44.6%), diabetes (19.1%) and other cardiovascular diseases (13.4%) were the most commonly reported comorbidities, and 22.2% presented severe/moderate signs of functional impairment.

The main diagnoses during hospitalization were malnutrition (76.0%) followed by depression (48.8%). Stroke and other cardiovascular diseases were found in 11.8 and 17.1% of hospitalized older adults, respectively. Thirty-eight (5.8%) older adults were HIV positive. Clinical characteristics of the study population is summarized in Table 2.

**Table 2:** Baseline clinical characteristics of hospitalized older adults in Togo in 2019 (*N* = 650)

	Total (***N*** = 650)	< 60 years (***N*** = 274)	≥ 60 years (***N*** = 376)	*p*-value
**Length of stay (days)**				0.085*
Median (IQR)	17 (8–32)	20 (10–34)	16 (8–32)	
**Type of hospitalization services, n(%)**				0.178**
Medical	494 (76.0)	201 (73.4)	293 (77.9)	
Surgical	156 (24.0)	73 (26.6)	83 (22.1)	
**Health pyramid level of hospital, n(%)**				0.739**
Tertiary	566 (87.1)	240 (87.6)	326 (86.7)	
Secondary	84 (12.9)	34 (12.4)	50 (13.3)	
**Comorbidities**				
**Diabetes, n(%)**	124 (19.1)	37 (13.5)	87 (23.1)	0.002**
**Hypertension, n(%)**	290 (44.6)	105 (38.3)	185 (49.2)	0.006**
**Other cardiovascular diseases, n(%)**	87 (13.4)	33 (12.0)	54 (14.4)	0.238**
**Osteoarthritis, n(%)**	51 (7.8)	16 (5.8)	35 (9.3)	0.104**
**Other comorbidities, n(%)**	24 (3.7)	6 (2.2)	18 (4.8)	0.511**
**Number of comorbidities, n(%)**				< 0.001**
0	262 (40.3)	131 (47.8)	131 (34.8)	
1	254 (39.1)	104 (38.0)	150 (39.9)	
≥ 2	134 (20.6)	38 (14.2)	95 (25.3)	
**Functional impairment, n(%)**				0.015**
None	506 (77.8)	226 (82.5)	280 (74.5)	
Moderate/Severe	144 (22.2)	48 (17.5)	96 (25.5)	
**Main diagnoses during hospitalization**				
**Malnutrition, n(%)**				0.003**
No	26 (4.0)	18 (6.4)	8 (2.1)	
Yes	494 (76.0)	204 (74.5)	290 (77.1)	
MD	130 (20.0)	52 (19.0)	78 (20.8)	
**Depression, n(%)**				0.440**
No	233 (35.8)	98 (35.8)	135 (35.9)	
Yes	317 (48.8)	145 (52.9)	172 (45.7)	
MD	100 (15.4)	31 (11.3)	69 (18.4)	
**HIV, n(%)**				0.001**
Negative	539 (83.0)	211 (77.0)	328 (87.2)	
Positive	38 (5.8)	26 (9.5)	12 (3.2)	
MD	73 (11.2)	37 (13.5)	36 (9.6)	
**Other cardiovascular diseases**^**a**^**, n(%)**	111 (17.1)	41 (15.0)	70 (18.6)	0.222**
**Other infections**^**b**^**, n(%)**	91 (14.0)	44 (16.1)	47 (12.5)	0.197**
**Stroke, n(%)**	81 (12.5)	28 (10.2)	53 (14.1)	0.174**
**Malignant diseases, n(%)**	75 (11.5)	29 (10.6)	46 (12.2)	0.516**
**Fractures and injuries, n(%)**	66 (10.2)	40 (14.6)	26 (6.9)	0.001**
**Gastrointestinal diseases, n(%)**	53 (8.2)	23 (8.4)	30 (8.0)	0.848**
**Renal diseases, n(%)**	49 (7.5)	16 (5.8)	33 (8.8)	0.161**
**Diabetes mellitus, n(%)**	31 (4.8)	6 (2.2)	25 (6.6)	0.008**

*MD* Missing data, *Wilcoxon-Mann-Whitney; **Chi square test

^a^Other cardiovascular diseases: Cardiomyopathy, congestive heart failure, hypertension

^b^Other infections: Rhinopharyngitis, Influenza and Pneumopathy

### All-cause mortality rate

A total number of 112 deaths occurred during the follow-up period, yielding an all-cause mortality rate of 17.2% (95%CI: 14.4–20.4). More than nine deaths in ten (93.7%; *n* = 105) occurred in hospital. There were 15.3% deaths among older adults < 60 years and 18.6% among those aged ≥60 years (*p* = 0.273). Overall mortality rate was 16.0 and 18.9% for male and female older adults, respectively (*p* = 0.337).

**Fig. 1:**
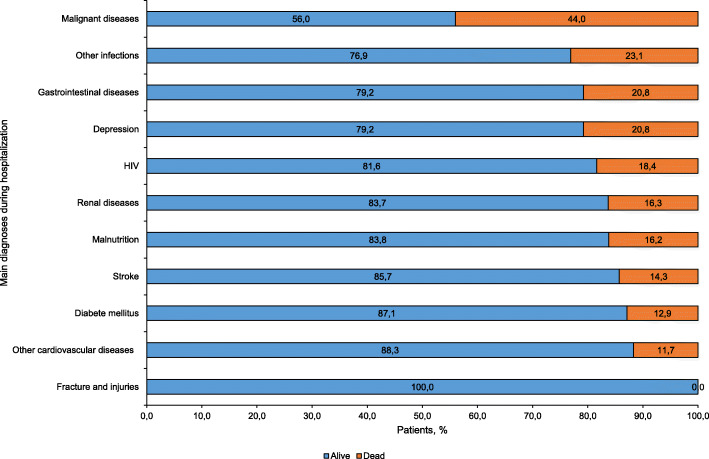
Main diagnoses during hospitalization by proportion of deaths

Figure 1 describes the main diagnoses during hospitalization by proportion of deaths. The highest proportions of death were observed in patients with malignant diseases (44.0%), other infections (23.1%), gastrointestinal diseases (20.8%) and depression (20.8%). The lowest proportions of death were observed in patients diagnosed with diabetes (12.9%) and other cardiovascular diseases (11.7%).

### Survival outcomes

**Fig. 2:**
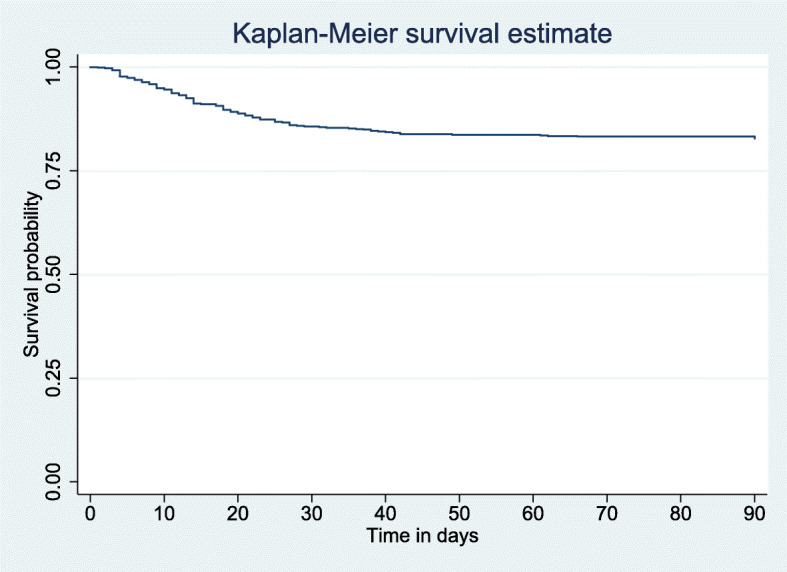
Overall survival curve of hospitalized older adults in Lomé, Togo in 2019

The main outcome measure was 3-month all-cause mortality. Kaplan-Meier estimates in each time period are shown in Fig. 2. The overall survival probability estimates were 0.911, 0.855, and 0.828 at 15, 30, and 90 days, respectively.

The probability estimates were 0.907, 0.840, and 0.811 for female and 0.921, 0.870 and 0.840 for male at 15, 30, and 90 days, respectively. According to age, these estimates were 0.931, 0.887, and 0.847 for older adults < 60 years and 0.896, 0.835, and 0.814 for those ≥60 years at 15, 30, and 90 days, respectively. There was no statistically significant difference in survival times according to sex (*p* = 0.340) and according to age groups (*p* = 0.280).

### Predictors of 3-month all-cause mortality

**Fig. 3:**
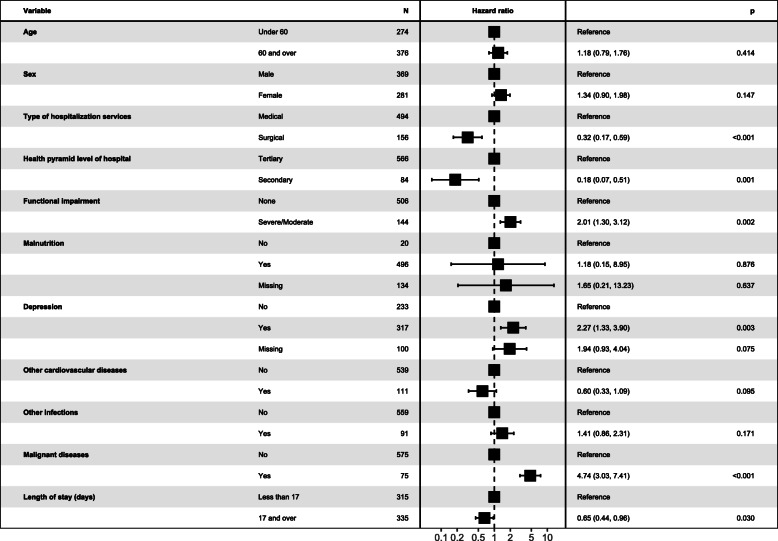
Predictors of 3-month all-cause mortality using Cox proportional hazard regression multivariable model (*N* = 650)

Figure 3 shows the predictors of 3-month all-cause mortality according to Cox proportional hazard regression multivariable model. Hospitalization in surgical services (HR = 0.23; 95%CI: 0.10–0.50), or in a hospital of the secondary level of the health pyramid (HR = 0.10; 95%CI: 0.03–0.43), and length of stay of 17 days and over (HR = 0.65; 95%CI: 0.44–0.96) were associated with lower risk of mortality within 3 month after hospitalization. Functional impairment (HR = 2.49; 95%CI: 1.48–4.21), depression (HR = 2.07; 95%CI: 1.16–3.70), and malignant diseases (HR = 4.15; 95%CI: 2.50–6.88) were significant predictors of 3-month all-cause mortality.

## Discussion

Among the 650 older adults who were recruited during the study period in selected hospitals, almost one fifth died within 3 months after their hospitalization. The majority of deaths recorded occurred during hospitalization and 16.2% of deceased older adults were malnourished. Overall survival rate was 82.8% after 90 days of follow-up. Factors associated with three-month mortality were the level of the hospital in the health pyramid, hospitalization service, length of stay, functional impairment, depression and malignant diseases.

Our study included both in-hospital and post-hospital mortality data. Deaths have been reported in almost one sixth of older adults, mostly during hospitalization. Similar findings have been reported in Africa. In a systematic review of the literature on hospital mortality among older adults in medical services in Africa which included five studies and 3427 older adults from Nigeria, Senegal, Morocco and Kenya, 22.6% of deaths have been reported [26]. Also, in South Africa, out of 11,254 older adults admitted to medical services during a four-year period, 15.1% of deaths were reported [9]. Mortality among hospitalized older adults in Africa is higher than that reported in more developed countries, suggesting poor quality of health care for older adults. For example, mortality among hospitalized older adults was 11.1% in the US [27], 8.4% in Turkey [8], and 8.2% in Taiwan [28]. Several reasons may explain these differences such as a more performant technical platform and a higher number of geriatricians. Indeed, in Africa, in 2012, there was no geriatrician in 23 countries, and 12 countries had 1 to 4 geriatricians [29] while the national ratio in Canada was 5 certified specialists in geriatric for 100,000 persons aged 65 years and older [30]. However, it should be noted that hospital mortality could be biased in favor of health facilities that have short hospital stays policy. Other reasons that could explain the high hospital mortality in Africa could be linked to the three “Ds”: “Delay in healthcare seeking”; “Delay in diagnosis” and “Delay in treatment”. In general, in Africa, the population does not consult a health professional when first symptoms appear. In most cases, people use traditional medicine or self-medicate and only consult when the condition becomes life-threatening [31, 32]. Also, most older adults in Africa do not have health insurance and they have to make out-of-pocket payments for healthcare services [33, 34].

Three hospital-related factors were associated with mortality in this study: the level of the hospital in the health pyramid, the service of hospitalization, and the length of stay. Older adults who were hospitalized in secondary level hospitals had lower risk of death compared to those hospitalized in tertiary level facilities. In fact, all severe cases are referred for treatment to tertiary level hospital. However, there is no geriatric services in these tertiary level hospitals while it has been reported that co-management of older adults with geriatric specialists is associated with decreased mortality [35]. We did not find any data in the literature that included health structures from different levels of the health pyramid in Africa. These results should be confirmed by further studies. Being hospitalized in surgical services was associated with lower risk of death for older adults. Given the risk of complications which are frequent after traumatic episodes such as fractures or surgical procedures [36], older adults are taken care of as soon as possible in surgical services, leading to the lower mortality observed.

Geriatric syndromes such as frailty, delirium, falls, dementia, depression, functional impairment, or malnutrition, have been consistently reported as factors of increased risk of death in several studies [10–17]. In our study, older adults with functional impairment or depression were twice as likely as those without these geriatric syndromes to die within the 3 months after hospitalization. We found no significant association between malnutrition and mortality. In a cohort study in Brazilian patients aged 65 years and older, the risk of death at 1 year was 5 times higher in malnourished patients [17]. Similar results were reported in Spain [14]. Geriatric syndromes can be quickly detected using validated tools tailored for older adults [23–25, 37] and most of them can be prevented, treated or managed appropriately [38–40]. For example, studies have reported that early nutritional intervention could reduce morbidity and mortality in hospitalized patients [38]. Screening for geriatric syndromes in the management of older adults is essential in order to provide them with appropriate health care.

The almost significant low risk of death in older adults hospitalized for other cardiovascular diseases (excluding stroke) was an unexpected result since cardiovascular diseases constitute significant risk factor for mortality, especially in older populations [41]. However, some traditional risk factors known to have a poor prognosis may vary in older adults and may contrast with expected effects. For example, in older adults, low BMI, low diastolic blood pressure and low cholesterol (which indicate reverse metabolic syndrome) have been shown to be significant predictors of mortality [42]. Future studies, such as prospective cohorts among older adults, are needed to confirm this result. In Italy, Ponzetto et al., studied risk factors affecting mortality in 987 patients aged ≥70 years admitted to a geriatric ward and reported that cerebrovascular diseases and cancer were health conditions associated with a high risk of hospital mortality [10]. In our study, the risk of death was four times higher in older adults diagnosed with cancer. In fact, in Togo, there is no dedicated oncology or radiotherapy department for the management of cancer in hospitals. Cancers are managed in organ-specific departments. For example, breast and lung cancers are managed in the gynecology and pulmonology departments, respectively. Furthermore, access to anti-cancer drugs is limited and sometimes not available in Togo [43].

Old age has been found to be a risk factor for mortality in hospitalized older adults because the likelihood of having a chronic disease or geriatric syndrome increases with age. However, age was not associated with mortality in our study. This difference can be explained by the age threshold fixed to define older adults. We included in our study patients aged 50 years and older as recommended by the WHO [44]. The majority of studies on older adults have included subjects at an older age from 60 years [9], or even 65 years in some studies conducted in developed countries [8]. Older women had a higher risk of death compared to older men, but the difference was not statistically significant (HR = 1.11; *p* = 0.579). These results contrast with that found in the literature where male sex was found to be associated with hospital mortality [10, 26, 45]. Reasons for gender-differences in mortality are that older women are more likely to seek healthcare and have healthier lifestyle [46]. Also, female hormones during fertile age may be protective for women with regards to cardiocirculatory events [46].

This study has some limitations. The main limitation is the non-documentation of causes of death due to the method of monitoring based on telephone contact. Abnormal laboratory tests results such as abnormal levels of hemoglobin, creatinine, urea, blood sugar have been found to be predictors of mortality in older adults [8, 26, 27]. For this study, we could not collect blood samples for laboratory testing due to the lack of financial means. Although deaths may be recorded in primary level structures, they were not selected for the study because these structures do not usually have hospitalization wards and are required to refer patients requiring hospitalization to tertiary and secondary level structures. Despite these limitations, to our knowledge, this study was the first to estimate mortality and to explore the associated risk factors in older adults after hospitalization in health facilities in Togo. The results of this study could help tailor strategies to the health needs of Togolese older adults.

## Conclusion

This study assessed mortality outcomes among older adults aged 50 years and older in Togo within the first 3 months after their admission to hospital. High hospital mortality rate has been reported, and the majority of deaths occurred during hospitalization. Mortality was associated to health structures-related factors, geriatric syndromes, cardiovascular diseases, and cancerous pathologies. The health system must adjust its response to an aging population in order to provide the most effective care.

## Supplementary Information

**Additional file 1.**

**Additional file 2.**

## Data Availability

All data used for the present study are available in the Supplementary File 2 (S2).

## Abbreviations

95% CI: 95% confidence interval
HR: Hazard ratio
CVD: Cardiovascular disease
IQR: Interquartile range
MD: Missing data
MNA-SF: Mini-Nutritional Assessment-Short Form
SSA: Sub-Saharan Africa
SHOT: Study of Hospitalized Older adults in Togo
WHO: World Health Organization.
