# Supplementary material for: Predictors of three-month mortality among hospitalized older adults in Togo
Source: BMC Geriatr. 2020 Nov 26;20:507. doi: 10.1186/s12877-020-01907-y (PMC7690011; doi:10.1186/s12877-020-01907-y)
Supplement: Supplementary file 1 — Additional file 1. [file 12877_2020_1907_MOESM1_ESM.pdf]

Study of hospitalized older adults in Togo

Installation: Patient seated or lying in supine position before reading note

Information note

In order to better structure the care of the older persons, the Department of Public Health is carrying out a study on the state of health of older persons hospitalized in health facilities in Togo. The information collected as part of this study will allow health strategies to be implemented to improve access to care and coordinate appropriate care targeting the specific features of older persons.

Have you been interviewed for this survey in the past few weeks?

❒ **No** ❒ **Yes**

If yes, tell him that you cannot include him in the study a second time, thank the participant and end the interview.

Your participation is voluntary and the data that will be collected will be anonymous (we will not mention your name or your first name). Try to respond as closely as possible to what fits your situation.

Suggest that the PA get help from someone who supports them in carrying out their health care and who knows them well (desirable but not compulsory).

We also ask you to do a urine collection to detect a urinary tract infection and a rapid diagnostic test for diabetes and HIV screening if they have not already been carried out during hospitalization. To the extent that there are free treatments for HIV / AIDS, it is to your advantage to know your results.

Do you agree to participate in the study (approximately 45 min interview)?

❒ **No** ❒ **Yes**

What's your birthday? Or your age?<br>National identity card presented?<br>☐ **No** ☐₁**Yes**<br><br>***Interview stopped if age less than 50***	| ___ | ___ | / | ___ | ___ | / | ___ | ___ | ___ | ___ | Day month Year<br>or<br>| ___ | ___ | years

a. Inclusion criteria

	Yes	No
1. Man or woman aged 50 or over?	☐1	☐2
2. Hospitalized in a service of selected health facilities?	☐1	☐2
3. Patient informed and having given his consent to participate in the study?	☐1	☐2

All previous boxes must be checked "yes" to include this patient in the study

b. Non-inclusion criteria

	Yes	No
1. Refusal or patient is unable to give consent	☐1	☐2
2. Severe neuropsychiatric disorders preventing somatic examination	☐1	☐2

All previous boxes must be checked "no" to include this patient in the study

Do you agree to perform the urine test? for diabetes screening? for HIV testing?

**Consent granted for urine test:** ·	❒ **No**	❒ **Yes**
**Consent granted for capillary blood sugar:**	❒ **No**	❒ **Yes**
**Consent granted for HIV testing :**	❒ **No**	❒ **Yes**

Signature or mark a cross by the older person or person designated by the older person (circle the appropriate mention):

Part 1: Initial face-to-face interview

Health structure	❒ Sylvanus Olympio University Hospital Center<br>❒ Campus University Hospital Center	❒ Kara University Hospital Center<br>❒ Others: ________________
Hospitalization service<br>(note the name of the department next to the specialty)	❒ Medicine<br>❒ Surgery	
ID number	| ___ | ___ | ___ || ___ | ___ | ___ || ___ | ___ || ___ | ___ | (First 3 letters Surname / first name, 2 initials of the hospital and last 2 digits of the year of birth)	
Survey date and start time	| ___ | ___ | / | ___ | ___ | / | ___ | ___ | ___ | ___ | | ___ | ___ | | ___ | ___ | Day month Year	
Date of admission to hospital	| ___ | ___ | / | ___ | ___ | / | ___ | ___ | ___ | ___ | Day month Year	
Surveyor's name		

Section 1: Socio-demographic characteristics

No.	Questions and referrals	Coded responses		Check
101	Sex	1. Male	2. Female	| ____ |
102	What level of education have you achieved?<br>***CIRCLE ONLY ONE ANSWER***	0. Has not been to school<br>1. Primary<br>2. Secondary	3. University<br>99. No response	| ____ |
103	What is your nationality?	1. Togolese	2. Other, specify: ______________	| ____ |
104	Do you live in Lomé?	0. No, specify ________________	1. Yes	| ____ |
105	What is your current marital status?	1. Married<br>2. Widow<br>3. Single	4. Divorced<br>5. Cohabitation<br>99. No response	| ____ |
106	Are you polygamous? (***for men only***)	0. No	1. Yes	| ____ |
107	How many wives do you have?	| __ | __ | women		| ____ |
108	How many children do you have in total?<br>(your own children)	| __ | __ | children<br>***MARK 00 IF NO CHILDREN AND*** → **110**		| ____ |
109	If you have children, how many are:	| __ | __ | living children?<br>| __ | __ | in the same city as you?	| __ | __ | in Togo (but not in the same city of residence)?<br>| __ | __ | abroad?	| ____ |
110	What is your current professional situation?	0. At retirement, no activities<br>1. Retired, exercising an income-generating activity	2. In activities<br>99. No response	| ____ |
111	Do you receive a pension?<br>***MULTIPLE ANSWERS POSSIBLE***	0. No<br>1. Retirement<br>2. Widowhood	3. Disability<br>4. Others, specify: ______________	| ____ |
112	Do you receive financial aid?<br>***MULTIPLE ANSWERS POSSIBLE***	0. No<br>1. Yes, children	2. Yes, close family<br>4. Others, specify: ______________	| ____ |
113	What is your main source of income?	0. None<br>1. Financial assistance<br>2. Pension	3. Income generating activities<br>4. Others, specify: ______________	| ____ |

114	In total, how much do you have per month to support yourself?	0. <10,000 FCFA<br>1. [10,000 to 35,000 [FCFA<br>2. [35,000 to 100,000 [FCFA	3. [100,000 to 150,000 [FCFA<br>4. [150,000 to 300,000 [FCFA<br>5. ≥ 300,000 FCFA	|____|
115	How many people do you have to support?	| __ | __ | people		|____|
116	Do you have health insurance?	0. No<br>1. Yes, INAM	2. Yes, private insurance	|____|
117	Which situation best describes your current housing conditions?	1. Owner<br>2. Tenant	3. For free with a loved one<br>4. Others, specify : _ ______________	|____|
11 8	You live	1. Alone<br>2. With spouse<br>3. Child	4. Brother or sister<br>5. Host family<br>6. Others, specify : _ ______________	|____|

Section 2: Reasons for hospitalization, history, lifestyle

**201**	What is (are) the main reason (s) for admission to hospital? (Reasons →·symptoms, transfer...)	1.<br>2.		|____|
**202**	What are the secondary diagnoses of hospitalization?	1.<br>2.		|____|
**203**	What is your medical and surgical history?	0. No history	8. Osteoarthritis / Rheumatism	
	(Several choices possible)	1. Hypertension	9. Dysthyroidism	|____|
	Check by looking in the medical file	2. Diabetes	10. Chronic obstructive pulmonary disease	
		3. Undernutrition	11. Dementia syndrome	
		4. Dyslipidemia	12. Cancer (specify):	
		5. Stroke	13. Others, please specify:	
		6. Arrhythmias	14. Others, please specify:	
		7. Myocardial infarction		
**204**	If hypertension, date of the last known follow-up consultation?<br>Otherwise → **207**	0. No 1. Yes<br>| ___ | ___ | / | ___ | ___ | / | ___ | ___ | ___ | ___ | Day month Year		|____|
**205**	If hypertension, do you have a drug treatment?<br>***(cross out the useless indication for the time unit)***	0. No	1. Yes<br>Since when? : | ___ | ___ | years / month	|____|
**206**	List these drugs<br>(International trade name)	1.<br>2.<br>3.		|____|
**207**	If diabetes, date of the last follow-up consultation?<br>Otherwise →↓**211**	0. No 1. Yes<br>| ___ | ___ | / | ___ | ___ | / | ___ | ___ | ___ | ___ | Day month Year		|____|
**208**	If you have diabetes, are you on medication?<br>***(cross out the useless indication for the time unit)***	0. No	1. Yes<br>Since when? : | ___ | ___ | years / month	|____|
**209**	List these drugs<br>(International trade name)	1.<br>2.<br>3.		|____|
**210**	If you have hypertension or diabetes, have you reduced your consumption			|____|
	- Of salt?<br>- Fatty foods?	0. No<br>0. No	1. Yes<br>1. Yes	

211	Do you currently smoke tobacco? (smoking cigarettes, cigars, pipes, chewing tobacco, snuff, others)	1. Yes, every day → **213**<br>2. Yes, but not every day → **213**<br>3. Don't smoke → **212**		|____|
212	**In the past,** have you smoked tobacco?	1. Yes, every day<br>2. Yes, but less than once a day	3. Not at all	|____|
213	In total, during or for how long have you smoked?	| ___ | ___ | years		|____|
214	How many days a week do you drink alcohol?	0. None → **215**<br>1. No more than 1 day per week	2. 2 to 3 days a week<br>3. More than 4 days a week	|____|
214	How much alcohol do you drink on a typical day when you drink? ***(several choices possible)***	0. Beer, one 33cl bottle = 1 dose<br>1. Beer, one 65 cl bottle = 2 doses<br>2. Liquor, one 25 ml glass = 1 dose<br>3. Local drink, a 500 ml calabash = 2 doses		|____|
215	During the week before your hospitalization, did you do any physical activity? *(tick one choice)*	0. No physical activity → **217**<br>1. Intense physical activity<br>2. Moderate physical activity		|____|
216	Specify number of sessions per week and the duration of each session:	| ____ | ____ | sessions / week 99. No answer<br>| ____ | ____ | minutes / session		|____|
217	During the week before your hospitalization, how many times did you walk for at least 10 minutes?	| ____ | ____ | sessions / week 99. No answer<br>| ____ | ____ | X | ____ | ____ | minutes / session 99. No answer		|____|
218	During the week before your hospitalization, how long did you spend in a sitting position?	| ____ | ____ | sessions / week 99. No answer<br>| ____ | ____ | X | ____ | ____ | minutes / session		|____|
219	How many servings of fruit or vegetables do you eat per day?	| ____ | ____ | portions		|____|
220	First blood pressure measurement in the right arm ***(report pulse)***	systolic blood pressure | ____ | ____ | ____ | mmHg<br>diastolic blood pressure | ____ | ____ | ____ | mmHg<br>Pulse 1:		|____|
221	Second blood pressure measurement on the left arm ***(report pulse)***	systolic blood pressure | ____ | ____ | ____ | mmHg<br>diastolic blood pressure | ____ | ____ | ____ | mmHg<br>Pulse 2:		|____|
222	Does anyone assist you during your hospitalization? (accompanying)	0. No	1. Yes	|____|
223	What are your ties to this person?	1. Housekeeper<br>2. Child<br>3. Mother / Father	4. Brother / Sister<br>5. Others, specify: ________________________	|____|

Section 3: Pain assessment

301	During this hospitalization :<br>- have you been asked if you are in pain?<br>- has the pain been assessed at least once?	0. No<br>0. No	1. Yes<br>1. Yes	| ____ |
302	Is verbal communication possible with the patient?	0. No → **304**	1. Yes	| ____ |
303	If yes, what is the intensity of the pain?<br>(***By using the slider of the ladder Visual Analogue***)	|___|___|/ **10** → **Q401**		| ____ |
304	Face: frown, grimaces, tightness, tight jaws, frozen face?	0. No	1. Yes	| ____ |
305	Look: inattentive, fixed, distant or begging, crying, eyes closed?	0. No	1. Yes	| ____ |
306	Complaints: "ouch", "ouch", "I'm in pain", screams, moans?	0. No	1. Yes	| ____ |
307	Body: withdrawal or protection of an area, refusal to mobilize, frozen attitudes?	0. No	1. Yes	| ____ |
308	Agitation, aggressiveness, grip?	0. No	1. Yes	| ____ |

Section 4: State of dependence and sensory state

	The following questions will allow us to determine your abilities to carry out daily activities such as dressing, washing or eating (within a week before hospitalization).				
		**Autonomous**	**Partial assistance**	**Dependent**	
**401**	To wash yourself	1	0.5	0	|___|
**402**	To dress	1	0.5	0	|___|
**403**	To go to the toilet	1	0.5	0	|___|
**404**	To move	1	0.5	0	|___|
**405**	For urinary elimination and defecation	1	0.5	0	|___|
**406**	To eat	1	0.5	0	|___|
**407**	Lawton scale in appendix completed?	0. No<br>If not why? _ ______________	1. Yes		|___|
**408**	Do you have visual impairment?	0. No	1. Yes		|___|
**409**	If yes, do you have corrective lenses?	0. No	1. Yes		|___|
**410**	Do you have hearing problems?	0. No → **501**	1. Yes		|___|
**411**	If yes, do you have hearing aids?	0. No	1. Yes		|___|

Section 5: Screening for cognitive impairment and depression

I'm going to ask you questions about your brief. Many older adults complain of temporary memory loss. The questions are sometimes difficult but also easy. (ST = subtotal). If not achieved, why?

**501**<br>**ST = 8**	***Which is :***	***Correct answers***		|___| **ST =**
	The day of the week?	0. No	1. Yes	
	The month of the year?	0. No	1. Yes	
	The season of the year?	0. No	1. Yes	
	Time of day?	0. No	1. Yes	
	Your age?	0. No	1. Yes	
	Your mother's name, first name?	0. No	1. Yes	
	Your personal address?	0. No	1. Yes	
	Where are we now?	0. No	1. Yes	
**502**<br>**ST = 6**	***I'll give you 6 words. I ask you, please the repeat after me.***			
	Car	0. No	1. Yes	|___| **ST =**
	Horse	0. No	1. Yes	
	Spoon	0. No	1. Yes	
	Peanut	0. No	1. Yes	
	Plane	0. No	1. Yes	
	Mango	0. No	1. Yes	
**503**<br>**ST = 2**	List the days of the week in reverse , starting with Sunday and then Saturday	0. No	1. Yes	|___| **ST =**
	A bowl of corn costs 200 FCFA. You give your daughter 1,000 FCFA. How many bowls of corn can she buy with this amount?	0. No	1. Yes	
**504**<br>**ST = 6**	***Can you please give me the 6 words that I gave you at the beginning of the interview? Try to keep them because I will ask for them later.***			
	Car	0. No	1. Yes	|___| **ST =**
	Horse	0. No	1. Yes	
	Spoon	0. No	1. Yes	
	Peanut	0. No	1. Yes	
	Plane	0. No	1. Yes	
	Mango	0. No	1. Yes	
**505**<br>**ST = 6**	***I will read you a short story. You will have to pay a lot of attention because I will only read it once. When I finish, I will wait a few moments and then ask you to tell me everything you remember about this story.***<br>“Three children were alone in a house. Suddenly, the fire started in the house. Their father managed to get in through the back window and get them out of the fire. Apart from a few minor injuries, the children feel very good. "			
	Three children in a house	0. No	1. Yes	|___|

	The house burned down	0. No	1. Yes	
	Their father managed to enter	0. No	1. Yes	ST =
	Children are saved	0. No	1. Yes	
	They have small wounds	0. No	1. Yes	
	They are fine	0. No	1. Yes	
506	***Could you remind me of the 6 words I gave you there has little time?***			
ST = 6	Car	0. No	1. Yes	|____|
	Horse	0. No	1. Yes	
	Spoon	0. No	1. Yes	
	Peanut	0. No	1. Yes	ST =
	Plane	0. No	1. Yes	
	Mango	0. No	1. Yes	
507	***I will ask you, please, to follow the following instructions and execute them as I will tell you.***			
ST = 3	Take the paper with the right hand	0. No	1. Yes	|____|
	Fold it in half with both hands	0. No	1. Yes	
	Drop it on the floor	0. No	1. Yes	ST =
508	***I'm going to show you things and ask you, please, to name them.***			
ST = 2	Watch	0. No	1. Yes	|____|
	Bracelet	0. No	1. Yes	ST =
509	Total points (number of "yes" from 501 to 508	|___|___|/|___|___|		|____|
510	Do you feel discouraged and sad?	0. No	1. Yes	|____|
511	Do you feel that your life is empty?	0. No	1. Yes	|____|
512	Are you happy most of the time?	0. No	1. Yes	|____|
513	Do you feel that your situation is hopeless?	0. No	1. Yes	|____|

If the total 509 < 28/39, give a coupon for neurological consultation and note the patient's identifier

Section 6 : Nutritional status

**601**	What was your weight before hospitalization? ***(999 if no answer)***	|___|___|___| **kg**		|____|
**602**	Patient in bed and can't get up?	0. No	1. Yes → **Q605**	|____|
**603**	Have you been weighed at least once in the hospital?	2. No<br>3. Yes → **note weight** |___|___|___| **kg**<br>**and weighing date** |___|___|/|___|___|/|___|___|___|___| Day Month Year		|____|
**604**	Patient's weight on the day of the survey	|___|___|___| **kg**	0. Not achievable	|____|
**605**	Known or measured size |___|___|___| **cm**	Knee-high heel |___|___|___| **cm**	0. Not achievable	|____|
**606**	Waist size	|___|___|___| **cm**	0. Not achievable	|____|
**607**	Calf circumference	0. <31 cm	1. > 31cm	
**608**	Does the patient have a loss of appetite? Has he eaten less in the past 3 months due to lack of appetite, digestive problems, difficulty chewing or swallowing?	0. Decreased food intake<br>1. Slight decrease in food intake	2. No drop in food intake	|____|
**609**	Recent weight loss (<3 months)	0. Weight loss> 3kg<br>1. Don't know	2. Between 1 and 3 kg<br>3. No loss	|____|
**610**	Mobility skills	0. In bed / in chair<br>1. Autonomous inside	2. Leaving the hospital room or room	|____|
**61 1**	Acute illness or psychological stress in the past 3 months?	0. Yes	2. No	|____|
**61 2**	Neuropsychological problem<br>***(to be completed by Dr GBEASOR)***	0. Dementia or severe depression<br>1. Mild dementia/depression	2. No psychological problem<br>99. ND	|____|

Section 7: Skin lesions and risk of bedsores

701	Presence of a skin lesion?	0. No	1. Yes	|____|
702	If skin lesion, what type (s) and seat (s) of the lesion?	1. Pressure ulcer<br>Seat 1: __________________<br>Seat 2: __________________<br>Seat 3: __________________	2. Ulcer<br>Seat 1: __________________<br>Seat 2: __________________<br>Seat 3: __________________	|____|
703	Another skin lesion?	0. No	1. Yes<br>Seat : ____________________	|____|
704	State General	a. Good<br>b. Average	c. Bad<br>d. Very bad	|____|
705	Mental state	a. Well<br>b. Apathetic	c. Confused<br>d. Unconscious	|____|
706	Autonomous activity	a. Without help<br>b. Help walking	c. Sitting in the chair Totally<br>d. bedridden	|____|
707	Mobility	a. Good<br>b. Decreased	c. Very limited<br>d. Motionless	|____|
708	Incontinence	a. No<br>b. Occasional	c. Urinary or fecal<br>d. Urinary and fecal	|____|

Section 8: Falls

801	How many times have you fallen	Last year? | __ | __ |	The last month? | __ | __ |	|____|
802	Can the patient stand up?	0. No → **901**	1. Yes → **803**	|____|
803	'Get up and g' test	1. Get up | __ |	4. Return to headquarters by doing the tour | __ |	|____|
		2. Walk to the wall | __ |	5. Sit down | __ |	|____|
		3. Turn around | __ |		|____|
		6. Total duration of the test | __ | __ | __ | seconds		|____|
804	Unipodal support held during	| __ | __ | seconds (left choice)	| __ | __ | seconds (right choice)	|____|

On your return, suggest carrying out screening for diabetes and then urinary tract infection. Make the capillary glycaemia before the urinary strip. During the urinary strip, complete the biological assessment in section 9 from the medical file.

Section 9: Hydration and biological balance

	If other units for the parameters, note the values under the section " Other units" specifying the units; NR : not requested; ND: not done			***Other units***	
901	Blood sugar (to be carried out during the survey) Specify whether on an empty stomach or not		| __ |. | __ | __ | **g / L**	☐ A fasting<br>☐No fasting	|____|
902	Thick smear	99. NR<br>88. ND	☐ Positive<br>☐ Negative		|____|
903	Hemoglobin level	99. NR<br>88. ND	| __ | __ |. | __ | __ | **g / dL**		|____|
904	Natremia	99. NR<br>88. ND	| __ | __ | __ | **mmol / L**		|____|
905	Kalemia	99. NR<br>88. ND	| __ | __ |. | __ | __ | **mmol / L**		|____|
906	Urea	99. NR<br>88. ND	| __ | __ |. | __ | __ | **mmol / L**		|____|
907	Creatinine	99. NR<br>88. ND	| __ | __ | __ | **mmol / L**		|____|
908	Albuminemia	99. NR<br>88. ND	| __ | __ | __ | **g / L**		|____|
909	TSH	99. NR<br>88. ND	| __ | __ |. | __ | __ | **mIU / L**		|____|
910	Liver enzymes<br>ASAT<br>ALAT	99. NR<br>88. ND<br>99. NR	| __ | __ | __ | __ | **U / L**<br>| __ | __ | __ | __ | **U / L**		|____|

		88. ND			
911	Urine dipstick performed?		0. No	1. Yes	|____|
912	**912 a** Urine dipstick results<br>***(mark the number of crosses)***		1. Leukocytes | __ | __ | cross<br>2. Nitrites | __ | __ | cross<br>3. Glycosuria | __ | __ | cross<br>4. Proteinuria | __ | __ | cross	**912 b**<br>Dysuria? 0. No 1. Yes<br>Burning during urination? 0. No 1. Yes<br>Lower back pain? 0. No 1. Yes	|____|
913	How much water do you drink per day?<br>(example : number of water bottles 1.5l ; or sachet of "pure water" = 500 ml)		| __ | __ |. | __ | __ | **L / day**		|____|

Give the results of the urine strip to the participant and explain if necessary an additional examination

For section 10, ask the elderly person if they would like the accompanying person to be present before continuing the investigation.

Section 10: HIV testing and care

**1001**	Have you ever had an HIV test?	0. No	1. Yes<br>99. Do not know	|____|
**1002**	During this hospitalization, have you ever taken an HIV test?	0. No → **1004**<br>1. Yes<br>99. Do not know →**1004**		|____|
**1003**	If yes, did you go back to get your test results?	0. No<br>1. Yes	99. Do not know	|____|
**1004**	Do you know of a treatment for people who have the AIDS virus (HIV)?	0. No	1. Yes	|____|
**1005**	Do you know the HIV status of your regular partner?<br>*If more than one partner, ask the question for the main partner.*	0. No	1. Yes	|____|
**1006**	Would you agree to be tested for HIV? (***if no go to Q1008)***	0. No	1. Yes	|____|
**1007**	If yes, would you like to know the result of your HIV test?	0. No	1. Yes	|____|
**1008**	If not why ?			|____|
**1009**	What is the result of the screening test?			|____|
**1010**	For men only: would you agree to have a digital rectal exam for prostate cancer screening?			|____|

Take the HIV test with the rapid diagnostic test and warn the patient of a waiting period for the results

Section 11: Treatment

**1101**	Do you have treatments?	0. No	1. Yes	|____|
**1102**	Which of the treatments you are taking have been prescribed by a doctor / health worker? Write the ITN (international trade name) of the drug<br><br>***Check the treatments prescribed during this hospitalization***<br><br>@		***Hospital***	|____|
		1.		
		2.		
		3.		
		4.		
		5.		
		6.		
		7		
		8.		
		9.		
		10.		

1103	Which of the treatments you take come from self-medication?	1.		|___|
		2.		
		3.		
		4.		
		5.		
1104	Have you used traditional treatments in the past 3 months?	0. No	1. Yes	|___|
1105	If yes, what types?	1. Plants<br>2. Scarifications	3. Others, specify: ______________________	|___|

Section 12: Household characteristics

1201	What type of house do you live in?	0. Common court	1. Individual house	|___|
1202	How many rooms are there in the house?	|__|__| rooms		|___|
1203	Do you have a car?	0. No	1. Yes	|___|
	A refrigerator?	0. No	1. Yes	
	A television?	0. No	1. Yes	
1204	Do you sleep on a mat?	0. No	1. Yes	|___|
	A bed?	0. No	1. Yes	
	Is your room air conditioned?	0. No	1. Yes	
	Do you have a fan in your room?	0. No	1. Yes	
1205	How many people sleep with you in your room?	|__|__| people		|___|
1206	Do you have an impregnated mosquito net?	0. No	1. Yes	|___|
	If you have any, do you sleep under impregnated mosquito net?	0. No, never<br>1. Rarely	2. Often<br>3. Always	
	Why don't you sleep under an impregnated mosquito net? (many answers possible)			
1207	What is your main means of travel?	0. Walking<br>1. Motorcycle (taxi)<br>2. Motorcycle (personal)	3. Car (personal)<br>4. Car (taxi)	|___|
1208	Do you have a housekeeper at home?	0. No	1. Yes	|___|

Give the results of the HIV test to the patient according to the methods validated in training and note to Q1009.

Would you like to be called back for a telephone follow-up within a month? ***0. No*** ***1. Yes***

Note the telephone numbers of the participant and the contact person if they wish to be contacted or if cognitive impairment is suspected.

Elderly number:

Referring number:

Interview end time: | ___ | ___ | | ___ | ___ |

NB: At the end of the initial face-to-face interview
1- Ask the interviewee if he has any questions
2- Check that all the questions are correctly filled
3- Thank the respondent
4- Give the neurology consultation coupon if suspected cognitive impairment
5- And send it for the additional blood sample to be taken if necessary

Part two: Telephone follow-up

Call the participant's contact number first

Section 13: Follow-up at 1 month

	Follow-up date at 1 month	| ___ | ___ | / | ___ | ___ | / | ___ | ___ | ___ | ___ | Day month Year			| ____ |
**1301**	Elderly person or contact person reachable?	1. Yes	2. No<br>Number of attempts:		| ____ |
**1302**	Is the elderly person	☐ Alive? ☐ Died?<br>If deceased, Date of death<br>| ___ | ___ | / | ___ | ___ | / | ___ | ___ | ___ | ___ |			| ____ |
**1303**	Is the elderly person	☐ Still hospitalized? ☐ Exit?<br>Hospital discharge date | ___ | ___ | / | ___ | ___ | / | ___ | ___ | ___ | ___ |			| ____ |
**1304**	From your hospitalization you will say that you have been	1. Very satisfied<br>2. Satisfied	3. Little satisfied<br>4. Dissatisfied		| ____ |
**1305**	If death, is the cause an accident on the public highway?	1. Yes	2. No		| ____ |
**1306**	If death, occurred in hospital	1. Yes	2. No		| ____ |
**1307**	How many times have you fallen since hospitalization at Hospital center?	| __ | __ | falls			| ____ |
**1308**	How many times have you been sick but not hospitalized since hospitalization at the hospital center?	| __ | __ | episodes of illness without hospitalization			| ____ |
**1309**	How many times have you been sick but not hospitalized since hospitalization at the hospital center?	| __ | __ | hospitalizations			| ____ |
	The following questions will allow us to determine your abilities to carry out daily activities such as dressing, washing or eating.				
		**Autonomous**	**Partial assistance**	**Dependent**	
**1310**	To wash yourself	1	0.5	0	| ____ |
**1311**	To dress	1	0.5	0	| ____ |
**1312**	To go to the toilet	1	0.5	0	| ____ |
**1313**	To move	1	0.5	0	| ____ |
**1314**	For urination and defecation	1	0.5	0	| ____ |
**1315**	To eat	1	0.5	0	| ____ |

Section 14: Follow-up at 3 months

	Follow-up date at 3 months	| ___ | ___ | / | ___ | ___ | / | ___ | ___ | ___ | ___ | Day month Year		| ____ |
**1401**	Elderly person or contact person reachable?	1. Yes	2. No<br>Number of attempts:	| ____ |
**1402**	Is the elderly person	☐ Alive? ☐ Died?<br>If deceased, Date of death<br>| ___ | ___ | / | ___ | ___ | / | ___ | ___ | ___ | ___ |		| ____ |
**1403**	If death, is the cause an accident on the public highway?	1. Yes	2. No	| ____ |
**1404**	How many times have you fallen since the initial hospitalization at the hospital center	| __ | __ | falls		| ____ |

	(***Specify date of initial interview)?***				
**1405**	How many times have you been sick but not hospitalized since the initial hospitalization at the hospital center (***specify date of initial interview)?***	| __ | __ | episodes of illness without hospitalization			|____|
**1406**	How many times have you been sick and hospitalized since the initial hospitalization at the hospital center (***specify date of initial interview)?***	| __ | __ | hospitalizations			|____|
	Determine your ability to perform daily activities such as dressing, washing or eating				
		**Autonomous**	**Partial assistance**	**Dependent**	
**1407**	To wash yourself	1	0.5	0	|____|
**1408**	To dress	1	0.5	0	|____|
**1409**	To go to the toilet	1	0.5	0	|____|
**1410**	To move	1	0.5	0	|____|
**1411**	For urination and defecation	1	0.5	0	|____|
**1412**	To eat	1	0.5	0	|____|

Section 15: Follow-up at 6 months

	Follow-up date at 6 months	|___|___|/|___|___|/|___|___|___|___| Day month Year			|____|
**1501**	Elderly person or contact person reachable?	1. Yes	2. No<br>Number of attempts:		|____|
**1502**	Is the elderly person	☐·Alive? ☐· Died?<br>If deceased, Date of death<br>|___|___|/|___|___|/|___|___|___|___|			|____|
**1503**	If death, is the cause an accident on the public highway?	1. Yes	2. No		|____|
**1504**	How many times have you fallen since the initial hospitalization at the hospital center (***specify date of initial interview)?***	| __ | __ | falls			|____|
**1505**	How many times have you been sick but not hospitalized since the initial hospitalization at the hospital center (***specify date of initial interview)?***	| __ | __ | episodes of illness without hospitalization			|____|
**1506**	How many times have you been sick and hospitalized since the initial hospitalization at the hospital center (***specify date of initial interview)?***	| __ | __ | hospitalizations			|____|
	The following questions will allow us to determine your abilities to carry out daily activities such as dressing, washing or eating.				
		**Autonomous**	**Partial assistance**	**Dependent**	
**1507**	To wash yourself	1	0.5	0	|____|
**1508**	To dress	1	0.5	0	|____|
**1509**	To go to the toilet	1	0.5	0	|____|
**1510**	To move	1	0.5	0	|____|
**1511**	For urination and defecation	1	0.5	0	|____|
**1512**	To eat	1	0.5	0	|____|

Section 16: 12 month follow-up

	12 month follow-up date	|___|___|/|___|___|/|___|___|___|___| Day month Year		|____|
**1601**	Elderly person or contact person reachable?	1. Yes	2. No<br>Number of attempts:	|____|
**1602**	Is the elderly person	☐ Alive? ☐ Died?<br>If deceased, Date of death<br>|___|___|/|___|___|/|___|___|___|___|		|____|
**1603**	If death, is the cause an accident on the public highway?	1. Yes	2. No	|____|

**1604**	How many times have you fallen since the initial hospitalization at the hospital center (***specify date of initial interview)?***	| __ | __ | falls			| ____ |
**1605**	How many times have you been sick but not hospitalized since the initial hospitalization at the CH (***specify date of initial interview)?***	| __ | __ | episodes of illness without hospitalization			| ____ |
**1606**	How many times have you been sick and hospitalized since the initial hospitalization at the hospital center (***specify date of initial interview)?***	| __ | __ | hospitalizations			| ____ |
	Determine your ability to perform daily activities such as dressing, washing or eating				
		**Autonomous**	**Partial assistance**	**Dependent**	
**1607**	To wash yourself	1	0.5	0	| ____ |
**1608**	To dress	1	0.5	0	| ____ |
**1609**	To go to the toilet	1	0.5	0	| ____ |
**1610**	To move	1	0.5	0	| ____ |
**1611**	For urination and defecation	1	0.5	0	| ____ |
**1612**	To eat	1	0.5	0	| ____ |
